# DNA methylation signature of psychological resilience in young adults: Constructing a methylation risk score using a machine learning method

**DOI:** 10.3389/fgene.2022.1046700

**Published:** 2023-01-12

**Authors:** Andrew Ke-Ming Lu, Shulan Hsieh, Cheng-Ta Yang, Xin-Yu Wang, Sheng-Hsiang Lin

**Affiliations:** ^1^ Institute of Clinical Medicine, College of Medicine, National Cheng Kung University, Tainan, Taiwan; ^2^ Department of Psychology, College of Social Sciences, National Cheng Kung University, Tainan, Taiwan; ^3^ Graduate Institute of Mind, Brain, and Consciousness, Taipei Medical University, Taipei, Taiwan; ^4^ Department of Public Health, College of Medicine, National Cheng Kung University, Tainan, Taiwan; ^5^ Biostatistics Consulting Center, National Cheng Kung University Hospital, College of Medicine, National Cheng Kung University, Tainan, Taiwan

**Keywords:** psychological resilience, psychology, epigenetics, DNA methylation, methylation risk score

## Abstract

Resilience is a process associated with the ability to recover from stress and adversity. We aimed to explore the resilience-associated DNA methylation signatures and evaluate the abilities of methylation risk scores to discriminate low resilience (LR) individuals. The study recruited 78 young adults and used Connor-Davidson Resilience Scale (CD-RISC) to divide them into low and high resilience groups. We randomly allocated all participants of two groups to the discovery and validation sets. We used the blood DNA of the subjects to conduct a genome-wide methylation scan and identify the significant methylation differences of CpG Sites in the discovery set. Moreover, the classification accuracy of the DNA methylation probes was confirmed in the validation set by real-time quantitative methylation-specific polymerase chain reaction. In the genome-wide methylation profiling between LR and HR individuals, seventeen significantly differentially methylated probes were detected. In the validation set, nine DNA methylation signatures within gene coding regions were selected for verification. Finally, three methylation probes [cg18565204 (AARS), cg17682313 (FBXW7), and cg07167608 (LINC01107)] were included in the final model of the methylation risk score for LR versus HR. These methylation risk score models of low resilience demonstrated satisfactory discrimination by logistic regression and support vector machine, with an AUC of 0.81 and 0.93, accuracy of 72.3% and 87.1%, sensitivity of 75%, and 87.5%, and specificity of 70% and 80%. Our findings suggest that methylation signatures can be utilized to identify individuals with LR and establish risk score models that may contribute to the field of psychology.

## Introduction

Resilience in the context of psychology is the process of successfully adapting or adjusting emotionally or socially despite being exposed to substantial stress, adversity, or trauma (Luthar et al., 2000; Rutter, 2006). Given the essential role of resilience, the study of its psychological basis has attracted much attention in past decades. Resilience is a complicated biopsychosocial phenomenon including genetic variations, epistasis, epigenetics, and gene-by-environment interactions. With recent advances in molecular genetics, elucidating the genetic basis of resilience has attracted increasing interest in the field of psychology (Wu et al., 2013; Mukherjee et al., 2014; Navrady et al., 2018). Identifying the characteristic genes or epigenetic markers would provide a clearer understanding of the biological and neuropsychological basis of resilience by helping to explain individual variability in resilience behaviors.

Previous studies suggested that genetics and epigenetics may influence the characteristics of resilience. The identification of characteristic genes may potentially explain individual differences in resilient behaviors and help explore the biological and neuropsychological basis of resilience. Six genes were found to be associated with resilience in previous studies: *BDNF*, *DRD4*, *5-HTTLPR*, *OXTR*, *CRHR1*, and *RGS2* (Niitsu et al., 2019). Epigenetic modifications are early events in stress resilience (Milaniak et al., 2017) and regulation of functional genes and regulatory sequences of synaptic plasticity in the brain and peripheral inflammation (Wang et al., 2018). Epigenetic mechanisms are one molecular pathway through which adverse and traumatic events become biologically embedded and lead to individual differences in resilience. Molecular profiling of the executive function of the human brain requires the full spectrum of molecular alterations that affect genome organization and subsequently the expression of genes in functionally mapped brain topologies (Egervari et al., 2019). This spectrum not only includes genetic defects but also epigenetic alterations, including DNA methylation. Numerous studies in the past have supported the hypothesis that adverse environments affect the epigenome and that epigenetic differences may discriminate susceptible from resilient subjects. DNA methylation of *MHC*, *DNMT3A*, *DNMT3B*, *NR3C1*, and *FKBP5* is significantly associated with posttraumatic growth, posttraumatic stress disorder, and resilience (Mehta et al., 2020; Miller et al., 2020). It is possible that epigenetic mechanisms underlie the complex etiology of anxiety disorders as well as the mechanisms that confer resilience (Schiele and Domschke, 2018). An animal study also demonstrated that aberrantly regulated DNA methylation mechanisms discriminatively underlie resilience and susceptibility to depression (Wang et al., 2018). The pattern of DNA methylation can be modulated by neuronal activity in response to physiological and environmental stimuli and is essential for brain function (Moore et al., 2013). A past study indicated that the blood–brain correlation (r) = 0.86 for methylation data averaged of each CpG across subjects and blood had the highest proportion (20.8%) of CpGs correlated to brain within human CpGs, as compared to buccal tissue (17.4%) and saliva (15.1%) (Braun et al., 2019). And other studies of psychiatric diseases have shown that the methylation pattern in the blood and the brain are potentially similar in some ways (Chen et al., 2017; Magwai et al., 2021). So, the altered methylation in the peripheral blood may reflect potential susceptibility biomarkers of resilience individuals. However, the mechanism of how methylation in peripheral blood affects brain resilience traits still needs further research in the future. The study using blood sample analysis also has the following advantages. First, DNA methylation in the blood are associated with early-life adversity and may represent a novel biomarker for early detection of psychopathology (Kundakovic et al., 2015). A previous study also found that a stressful environment can alter methylation patterns in blood (Murgatroyd et al., 2009). Second, in clinical collections, blood is a suitable tissue because it is easy to collect and the specimen is relatively stable. Third, if the population is to be widely used in the future, the method of blood collection has relatively little safety risk to the participants (Clark et al., 2020).

Epigenetic mechanisms are hypothesized as a molecular pathway for how stressful events lead to differences in individual resilience. Resilience research is needed to prevent mental disorders and promote competent, healthy development. Both optimism and skepticism have been expressed about the capability of current research to aid this objective (Kim-Cohen and Gold, 2009). However, not much is known about the role of DNA methylation in the development of psychological resilience. DNA methylation is a stable epigenetic mark and some methylation patterns may be preserved as a form of epigenetic memory (Kim and Costello, 2017; Li et al., 2022). The study aim was to explore DNA methylation signatures associated with low resilience and establish methylation risk scores by machine learning. Also, we hope that DNA methylation signatures may potentially be used to generate discriminative algorithms to improve mental health.

## Materials and methods

### Study subjects

We recruited 20- to 30-year-old participants from southern Taiwan. All participants were divided into two groups—subjects with low resilience (LR) and high resilience (HR)—using the Connor–Davidson Resilience Scale (CD-RISC) and randomly assigned to the discovery and validation sets. In this study, high resilience and low resilience individuals were matched with age and gender. In the discovery set, 8 HR individuals (CD-RISC score ≥60 out of 100) and 8 LR individuals (CD-RISC score <60) were randomly selected for genome-wide methylation profiling used to detect significant methylation markers. The methylation signatures were verified in the validation set (31 LR individuals and 31 HR individuals). We excluded participants that 1) had shrapnel or other metal or electronic implants in their bodies (such as aneurysm clips, pacemakers, surgical devices, or metallic tattoos on the head); 2) were pregnant or breastfeeding; 3) had a history of head trauma or surgery; or 4) had major medical, neuropsychiatric, or psychological disorders (including depression, generalized anxiety, panic attacks, ADHD, claustrophobia, strokes, heart conditions, tumors, or substance abuse). Data were collected from September 2020 to December 2021. The study received ethical approval from the institutional review boards (IRB) of National Cheng Kung University Hospital. Written informed consent was obtained from all study subjects.

### Psychological measurements

In this study, we used the 25-item self-assessment of the Connor-Davidson Resilience Scale (CD-RISC). This scale uses a 5-point scale (0–4) to assess resilience, defined as the ability to bounce back after stressful events, tragedy and trauma, based on previously identified characteristics shared among resilient subjects (Connor and Davidson, 2003). The total score is from 0 to 100, with higher scores indicating higher resilience. The CD-RISC shows good internal consistency (Cronbach’s α = 0.89), test-retest reliability (intraclass correlation coefficient, ICC = 0.87), and convergent validity with the Perceived Stress Scale (Pearson’s r = −0.76, *p* < .001) (Connor and Davidson, 2003).

Another psychological assessment we used was the 21-item Beck Depression Inventory-II (BDI-II) to measure the presence and severity of depressive symptoms (Beck et al., 1996). The assessment uses a self-reported 4-point scale (0–3) to detect depression symptoms and the severity of each reported symptom. The BDI-II evaluates the affective, behavioral, and somatic symptoms of depression. The assessment shows a test-retest reliability of 0.93 and split-half internal consistency reliability of 0.91 (Beck et al., 1996).

### Collection of blood sample and DNA extraction

EDTA (ethylenediaminetetraacetic acid) blood was collected from the forearm veins of all participants. Within 2 h of collection, blood samples were separated into plasma, buffy coat, and red blood cells by centrifugation at 800 × *g* for 10 min at 4°C. The samples were then placed in RNase/DNase-free microcentrifuge tubes and stored at −80°C. Following the manufacturer’s instructions, DNA was extracted from buffy coat samples by the QIAamp DNA Mini Kit (Qiagen, Germany). Integrity, quantity, purity, and concentration was assessed by UV–Vis spectrophotometer (NanoDrop 2000, NanoDrop, United States) and electrophoresis in a 1% agarose gel.

### Genome-wide DNA methylation profiling, quality control, and normalization

For global methylation profiling, the study used the Illumina Infinium Human Methylation 850 BeadChip (Illumina, United States), which interrogates DNA methylation status of >850,000 CpG methylation sites. The CpG sites were identified in the genome-wide DNA methylation analysis and mapped on the genome using the UCSC genome browser. We performed bisulfite conversion on 500 ng of genomic DNA from every samples by the EZ DNA Methylation Kit (D5002, Zymo Research, United States) based on the manufacturer’s standard protocol. Bisulfite-converted DNA was whole-genome amplified and enzymatically fragmented prior to hybridization to BeadChip arrays. The Illumina iScan Reader was utilized to scan arrays (Illumina, United States). Specimens were analyzed for global patterns of DNA methylation, after which DNA methylation was quantified on candidate differentially methylated CpG units and confirmed in study samples. The image files (IDATs) of the study were processed into R software ChAMP package for handling Illumina methylation array data (Maksimovic et al., 2016). The level of DNA methylation at each CpG locus was given a beta value, which was calculated as (M/(M + U)) and ranged from 0 to 1. Values close to 1 indicated that there was high methylation, while values close to 0 indicated that there was low methylation. Quantile normalization was performed before detecting differentially methylated CpG sites. Differentially methylated probes were identified based on three criteria: the average-Nbeads of probes were ≥3 in the samples; probe detection was at a significance level of *p* ≤ .05; and the β-value difference between the two groups was ≥ 0.25. The differential methylation quantification was required because only the information of representative CpG methylation states was provided by human methylation microarray methylomes.

### Real-time quantitative methylation analysis

To validate the methylated regions identified by quantitative methylation-specific PCR (qMSP) assay was carried out with Kapa Sybr Fast qPCR kit (Kapa Biosystems, United States) at the StepOnePlus Real-Time PCR System (Thermo Fisher Scientific, United States). The target genes of primer and probe sequences were designed in Supplementary Table S1. We used the following formula: 2 [Ct (β-actin)—Ct (candidate)] × 100 to estimate the methylation level as the difference in Ct value between β-Actin and the target candidates (Hesselink et al., 2011; Lu et al., 2017).

### Statistical analysis

All analyses were performed using R v4.1.2 (R Foundation, Vienna, Austria) and SAS v9.4 (SAS Institute Inc., NC, United States). Demographic characteristics were expressed as the mean ± standard deviation (SD) for continuous variables and presented as percentages for categorical variables. Student’s two-sample *t*-test was used for continuous data and Pearson’s chi-square test was used for categorical variables to assess the difference for group comparisons.

For distinguishing between LR and HR individuals by the methylation probes, we applied the receiver operating characteristic (ROC) curve analysis generated using logistic regression and support vector machine (SVM). Use the principle of statistical risk minimization to estimate a classification hyperplane, and find a decision boundary to maximize the margins between the two groups (Mustafa et al., 2018).

In the methylation risk score model, differentially methylated signatures between LR and HR were used to weighting by logistic regression and SVM and the coefficient estimates of the methylation signatures were then calculated. The methylation risk score was expressed as $\overset{n}{\underset{i=1}{\sum }}b_{i}x_{i}$ , where x represented the levels of methylation signatures (Deng et al., 2020). The LR and HR individuals were separated according to the logistic regression based on the methylation risk score under the hypothesis that the risk more might constitute a suitable discriminator. To assess associations among the methylation probes, methylation risk score, CD-RISC and BDI-II, we examined correlation matrices based on Spearman’s rank-order correlation coefficients.

### Enrichment analysis

In this study, we used the Enrichr website (Xie et al., 2021) for enrichment analysis. There are many databases in this website, which can present the analysis results of many different diseases or mechanisms. We primarily present the results of our analysis for neuropsychiatric disorders based on we hypothesized that low resilience may be associated with neuropsychiatric disorders by the previous literature review. In our recruited participants all excluded neuropsychiatric disorders, such that may be possible to illustrate the potential relationship between low resilience and neuropsychiatric disorders. In addition, we used the PheWeb database from the Enrichr website because it contains a lot of information about psychiatric and neurological disorders.

## Results

### Demographic characteristics of study participants

The demographic data of the participants (76 young adults, ages ranging from 20 to 30 years old) was shown in Table 1. We split all participants into a discovery and a validation sets. The distribution of age, gender, education, BMI, smoking, alcohol drinking, CD-RISC, and BDI-II is compared between individuals with low resilience (LR) and high resilience (HR). In this study, CD-RISC scores ≥60 out of 100 were defined as HR and CD-RISC scores <60 out of 100 were defined as LR. The CD-RISC and BDI-II scores were significantly different between the two groups (*p* < .001). The age, gender, education, BMI, smoking, and alcohol drinking variables were not significantly different between the two groups.

**TABLE 1:** Demographics and clinical characteristics of LR and HR groups.

Variables	Discovery set					Validation set				
	Low resilience		High resilience			Low resilience		High resilience		
	(N = 8)		(N = 8)		*p*-value	(N = 31)		(N = 31)		*p*-value
	N	%	N	%		N	%	N	%	
Male	4	50	4	50		15	48.3	16	51.6	
Smoking	0	0	0	0		0	0	0	0	
Alcohol drinking	0	0	0	0		0	0	1	0.0	
	Mean	SD	Mean	SD		Mean	SD	Mean	SD	
Age (years)	21.5	1.41	22	2.14		22.7	2.3	22.6	3.0	
Education (years)	16.3	2.0	16.1	1.7		16.4	2.2	15.9	2.0	
BMI (kg/m2)	24.7	1.6	21.6	1.2		24.0	5.8	22.5	4.2	
CD-RISC	38.9	14.0	91.0	4.7	a	49.9	7.3	78.2	7.0	a
BDI-II	17.9	8.6	7.8	8.7	a	11.3	8.3	4.9	3.2	a

LR, low resilience; HR, high resilience; SD, standard deviation; BMI, body mass index; CD-RISC, Connor-Davidson resilience scale; BDI-II, Beck depression inventory-II.

^a^ significant difference between LR and HR, *p* < .05.

### Genome-wide DNA methylation profiling in the discovery set

**FIGURE 1:**
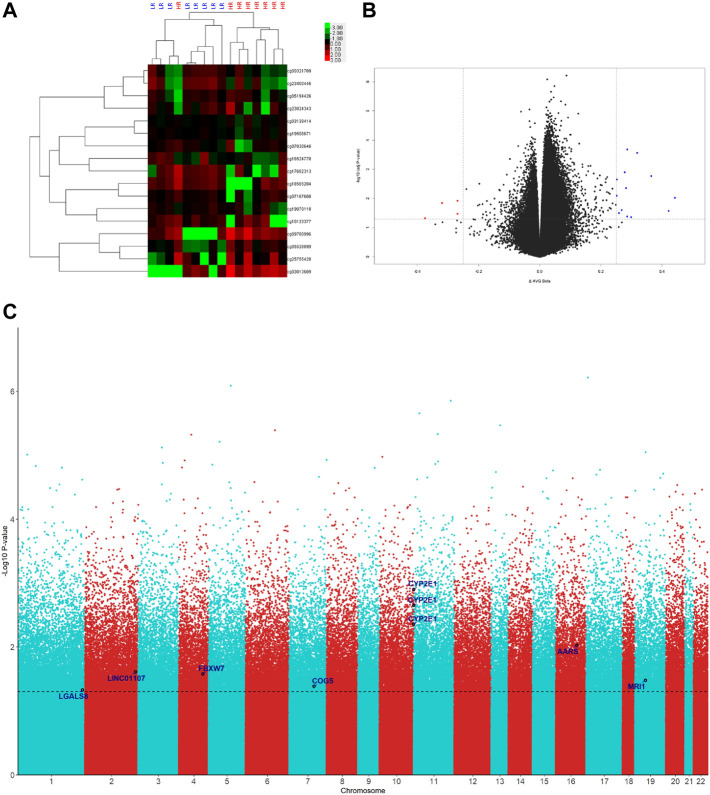
Genome-wide DNA methylation significantly associated probes of LR (low resilience) versus HR (high resilience) groups in the discovery set. **(A)** Heatmap of differentially methylated probes with >25% differences and *p* < .05 between LR and HR. **(B)** Volcano plot of differentially methylated probes with >25% differences and *p* < .05 between LR and HR. **(C)** Manhattan plot of differentially methylated probes with >25% differences and *p* < .05 between LR and HR.

The levels of DNA methylation were compared between the LR and HR groups (eight subjects each) in the discovery set by Human Methylation 850 BeadChip platform (Illumina). The difference criteria were a *β*-Value difference between two groups ≥.25 and *p*-Value <.05 (Figures 1A, B). A total of seventeen differentially methylated probes were detected (Table 2). The gene list includes *CYP2E1*, *AARS*, *LINC01107*, *FBXW7*, *MRI1*, *COG5*, and *LGALS8* (Figure 1C). Therefore, we selected nine signatures within coding regions for these genes for confirmation in the validation set.

**TABLE 2:** Genome-wide DNA methylation probe levels in the LR and HR groups of the discovery set^a^.

Probe ID	Mean *β* values in LR	Mean *β* values in HR	*p*-value	Gene
cg03139414	.893180042	.607209185	.000207411	
cg19508671	.822328634	.504008435	.000271561	
cg00321709	.458138739	.180541564	.001256527	*CYP2E1*
cg19070118	.777427782	.412867498	.001690978	
cg23400446	.383165974	.131292967	.00223429	*CYP2E1*
cg05194426	.616399879	.334172033	.004403253	*CYP2E1*
cg16524778	.425451486	.173924232	.008112836	
cg18565204	.972945459	.53091779	.009455589	*AARS*
cg05528899	.690132696	.958883762	.011982008	
cg09780996	.304721816	.624051331	.014250895	
cg07167608	.841830328	.57399626	.024667224	*LINC01107*
cg17682313	.834110455	.412761164	.02643509	*FBXW7*
cg07030646	.781571158	.522591259	.031194558	
cg25755428	.309003001	.577580963	.033384153	*MRI1*
cg23024343	.747953956	.462397392	.041271331	*COG5*
cg10123377	.736455899	.437780254	.043007361	
cg03013609	.390040555	.764941446	.047209222	*LGALS8*

LR, low resilience; HR, high resilience.

^a^ Methylation probe levels with greater than 25% mean methylation differences with *p*-value< .05 between LR and HR in the discovery set.

### Identification of DNA methylation signatures in the validation set

**FIGURE 2:**
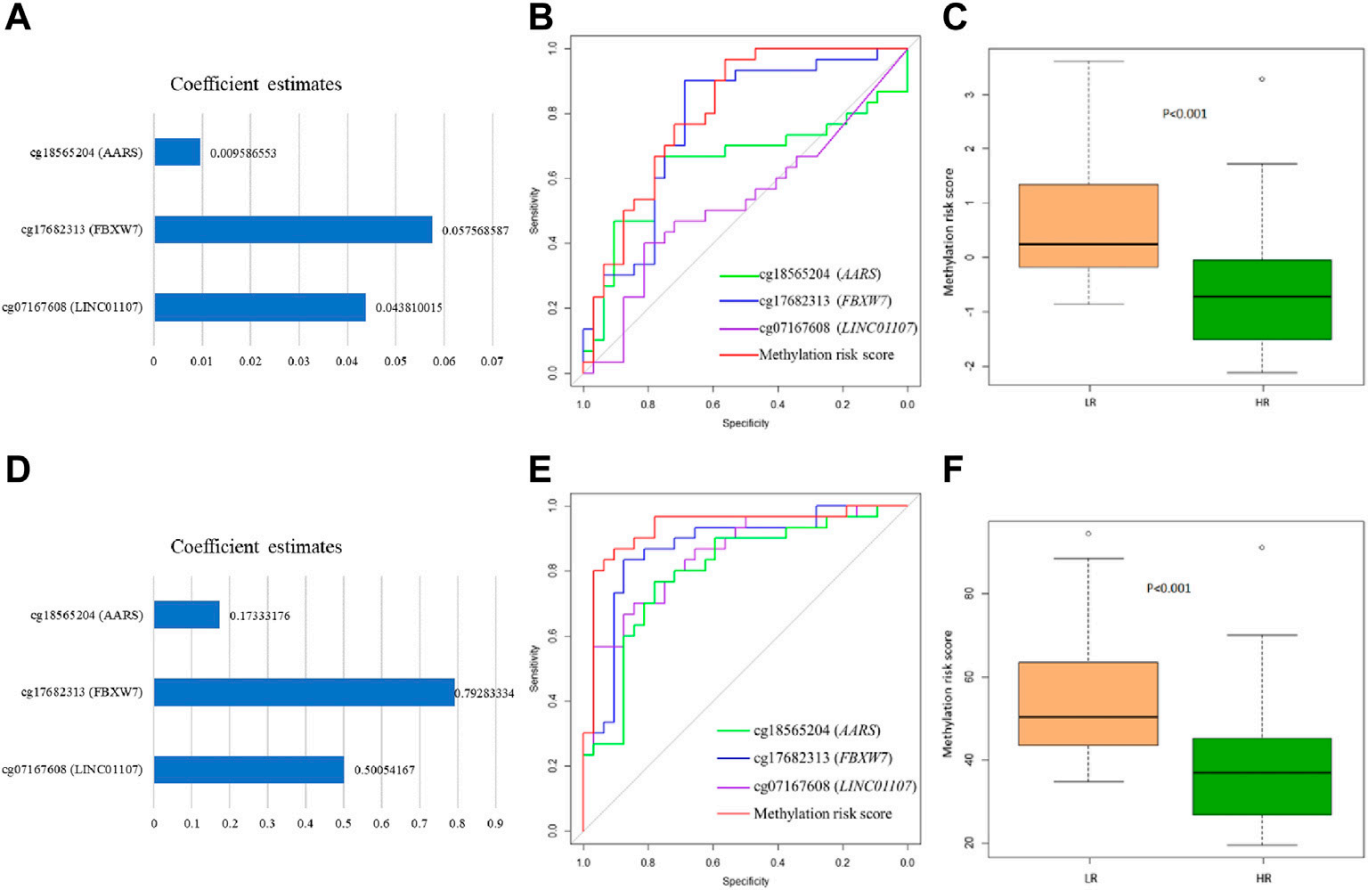
Receiver operating characteristic (ROC) curves and forest plot of methylation probes and methylation risk score using logistic regression and support vector machine in LR (low resilience) versus HR (high resilience) groups in the validation set. **(A)** Forest plot showing methylation probe coefficient estimates for methylation risk scores by logistic regression. **(B)** ROC curves showing the capabilities of logistic regression to discriminant LR. **(C)** Box plot showing methylation risk scores of the three methylation probes in LR and HR by logistic regression. **(D)** Forest plot showing methylation probe coefficient estimates for methylation risk scores by support vector machine. **(E)** ROC curves showing the capabilities of support vector machine to discriminant LR. **(F)** Box plot showing methylation risk scores of the three methylation probes in LR and HR by support vector machine.

The mean level of the nine methylation signatures was summarized in Supplementary Table S2. There were significant differences in cg18565204 (*AARS*), cg17682313 (*FBXW7*), and cg07167608 (*LINC01107*) between two groups. We also conducted linear regression to explore possible predictors of methylation probe levels. There were two identified probes had significant effects (*p* < .05) and one identified probe had marginal significant effects (*p* < .06) as predictors in the model, as shown in Supplementary Table S3. Furthermore, the linear regression model for adjusted gender and age was shown in Supplementary Table S4. After adjusting for gender and age, the results were similar to the unadjusted results. We calculated the area under the curve of ROC curve analysis by logistic regression and SVM to estimate the discrimination ability of DNA methylation signatures between LR and HR. The results were shown in Table 3. The best methylation probe discriminated between LR subjects and HR subjects was cg17682313 (*FBXW7*) by logistic regression and support vector machine, with an AUC of 0.77 and 0.87, accuracy of 71% and 83.9%, sensitivity of 72% and 86.7%, and specificity of 70% and 81.3%. The derived receiver operating characteristic (ROC) curve was shown in Figures 2B, E.

**TABLE 3:** Logistic regression and SVM models of methylation probes and methylation risk scores for LR and HR groups in the validation set.

	Logistic regression				SVM			
Variables	AUC (95% CI)	Accuracy (%)	Sensitivity (%)	Specificity (%)	AUC (95% CI)	Accuracy (%)	Sensitivity (%)	Specificity (%)
cg18565204 (*AARS*)	**.65 (.50, .80)**	**62.9**	**70**	**56.2**	**.80 (.69, .91)**	**75.8**	**78.7**	**75**
cg17682313 (*FBXW7*)	**.77 (.65, .89)**	**71**	**72**	**70**	**.87 (.78, .97)**	**83.9**	**86.7**	**81.3**
cg23024343 (*COG5*)	.47 (.31, .60)	43.6	50	37.5	.63 (.49, .77)	66.1	76.7	56.3
cg05194426 (*CYP2E1*)	.50 (.35, .64)	50	50	50	.77 (.66, .89)	69.4	86.7	53.1
cg25755428 (*MRI1*)	.54 (.39, .68)	50	50	50	.78 (.67, .90)	69.4	88.3	56.3
cg23400446 (*CYP2E1*)	.54 (.40, .69)	56.5	60	53.1	.78 (.67, .89)	67.7	87.3	53.4
cg07167608 (*LINC01107*)	**.73 (.61, .86)**	**71.0**	**73.3**	**68.8**	**.85 (.76, .97)**	**75.8**	**83.7**	**61.3**
cg03013609 (*LGALS8*)	.53 (.38, .68)	51.6	53.3	50	.78 (.67, .89)	72.6	86.7	59.4
cg00321709 (*CYP2E1*)	.53 (.38, .67)	54.8	60	50	.78 (.67, .90)	69.4	80	59.4
Methylation risk score (cg18565204 (*AARS*) + cg17682313 (*FBXW7*) + cg07167608 (*LINC01107*))	**.81 (.72. .90)**	**72.3**	**75**	**70**	**.93 (.86, 1)**	**87.1**	**87.5**	**80**

SVM, support vector machine; LR, low resilience; HR, high resilience; AUC, area under the ROC curve.

Bold values denote good discriminant ability.

### Methylation risk score of LR individuals in the validation set

We selected three probes (cg18565204, cg17682313, and cg07167608) that showed good discrimination to establish methylation risk scores by two statistical methods (logistic regression and support vector machine). In the logistic regression model, the coefficient estimates of DNA methylation signatures of the methylation risk score model were showed in Figure 2A. This risk score of the subjects ranged from −2.11 to 3.61 in the validation set. We split the subjects into LR and HR groups by a cut-off of ≥−0.59; the optimal cut-off value was calculated using the ROC curve analysis. We found significant differences in methylation risk scores (Figure 2C) between LR and HR subjects (*p* < .001). In the support vector machine model, the coefficient estimates of DNA methylation signatures were showed in Figure 2D. This risk score of the subjects ranged from 19.51 to 94.41 in the validation set. We split the subjects into LR and HR groups by a cut-off of ≥39.96. Significant differences in methylation risk scores (Figure 2F) could distinguish between LR and HR subjects (*p* < .001). The results showed that greater risk scores were associated with low resilience based on the corresponding risk score value. These methylation risk score models (cg18565204 + cg17682313 + cg07167608) showed good discrimination between LR and HR subjects by logistic regression and support vector machine, with an AUC of 0.81 and 0.93, accuracy of 72.3% and 87.1%, sensitivity of 75% and 87.5%, and specificity of 70% and 80%. The results of ROC curve analysis in the methylation risk score were shown in Figures 2B, E.

### Associations between CD-RISC, BDI-II, and DNA methylation signatures

CD-RISC can be divided into five factors: personal competence, trust, acceptance, control, and spirit (Connor and Davidson, 2003). The association between these five factors of resilience and the estimated metrics (methylation signatures level and methylation risk score) was investigated using the Spearman rank-order correlation test; the correlation plot is displayed in Supplementary Figure S1. The cg17682313 showed negative correlations with total score of CD-RISC (r_s_ = −.456, *p* < .001), personal competence (r_s_ = −.360, *p* < .01), trust (r_s_ = −.424, *p* < .001), acceptance (r_s_ = −.304, *p* < .05), control (r_s_ = −.429, *p* < .001), and spirit (r_s_ = −.355, *p* < .01) in all individuals. The cg07167608 showed negative correlations with total score of CD-RISC (r_s_ = −.266, *p* < .05), trust (r_s_ = −.390, *p* < .01), and control (r_s_ = −.261, *p* < .05) in all individuals. The methylation risk score showed negative correlations with total score of CD-RISC (r_s_ = −.459, *p* < .001), personal competence (r_s_ = −.375, *p* < .01), trust (r_s_ = −.494, *p* < .001), acceptance (r_s_ = −.363, *p* < .01), control (r_s_ = −.450, *p* < .001), and spirit (r_s_ = −.306, *p* < .05) in all individuals. Equivalent tests of the association between CD-RISC, BDI-II and the same metrics are shown in Supplementary Figure S2.

### Enrichment analysis of functional annotation in networks of individuals with LR

We generated a gene-gene enrichment analysis on PheWeb databases by the coding genes (*AARS*, *FBXW7*, and *LINC01107*) for the three methylation probes of the methylation risk score model in the validation set. The network analysis focuses on the related diseases. The gene sets were associated with some neuropsychiatric diseases and other neuronal functions including bipolar disorder (*p* < .01), schizophrenia (*p* = .01), circadian rhythm (*p* = .02), brain (*p* = .02), intelligence tests (*p* = .04), child development disorders (*p* = .05), autistic disorder (*p* = .07), depressive disorder (*p* = .07), attention deficit disorder (*p* = .08), and Alzheimer’s disease (*p* = .15). A Manhattan plot of the enrichment analysis was shown in Supplementary Figure S3.

## Discussion

We performed three steps for a DNA methylation analysis of the study. According to the results, a total of seventeen CpG sites that were differentially methylated between the LR and HR cohorts were identified in the discovery set. The nine methylation signatures within gene coding regions were selected for confirmation in the validation set. Finally, the study selected three methylation probes that were significantly effective differentiators to build the methylation risk score model for low resilience. These three methylation probe coding genes were associated with neuropsychiatric diseases. To our knowledge, this study is the first to investigate DNA methylation in resilience and to develop methylation risk score models for low resilience using logistic regression and machine learning methods.

In this study, three probes within relevant gene coding regions (*AARS*, *FBXW7,* and *LINC01107*) were identified. These genes also overlap with some brain function and neuropsychiatric disease-associated genes. *AARS* (human alanyl-tRNA synthetase) belongs to a family of tRNA synthases of the class II enzymes (Rajendran et al., 2018). Multiple paths of the evidence (that is tRNA gene mutations, tRNA epitranscriptome, tRNA charging, and tRFs) support that various aspects of tRNA function and metabolism are linked to neurodevelopmental and neuropsychiatric disorders (Legge et al., 2019; Blaze and Akbarian, 2022). *FBXW7* encodes a member of the F-box protein subunit of an Skp1-Cul1-F-box protein (SCF)-type ubiquitin ligase complex and plays a principal role in the degradation of Notch family members (Snyder et al., 2012). *FBXW7* is also an important regulator of the maintenance and differentiation of neural stem cells in the brain (Matsumoto et al., 2011). The gene was associated with neurodevelopmental syndromes and is distinguished by global developmental delays, borderline to severe intellectual disability, hypotonia, and gastrointestinal functions (Stephenson et al., 2022). *LINC01107* (Long Intergenic Non-Protein Coding RNA 1107) is an RNA gene affiliated with the lncRNA class. However, the gene set in our final selection (*AARS*, *FBXW7*, and *LINC01107*) was associated with brain functions, psychiatric disease and neurological disease. This means that we identified methylation of resilience-associated genes that may play a potential role in neuropsychiatric diseases. In addition, our enrichment analysis was found that this gene set are associated with some neuropsychiatric diseases and other neuronal functions including bipolar disorder, schizophrenia, circadian rhythm, brain, intelligence tests, child development disorder, autistic disorder, depressive disorder, attention deficit disorder, and Alzheimer’s disease. This result may explain the association between methylation abnormalities of low resilience individuals and brain, neurological, and psychiatric disorders, but the causation and mechanism must be confirmed. In the enrichment analysis, we also found some results on non-neuropsychiatric disorders. We are currently unable to explore the relationship between resilience and these physical diseases because we did not exclude these diseases from all participants. Furthermore, the findings of association between the methylation signatures and psychological resilience still need further research to parse out the biological mechanism.

The findings of this study link altered expression of DNA methylation signatures to biological pathways involved in resilience; however, there are some limitations in the study. First, resilience may dynamically change during development and time since exposure (Windle, 2011; Stainton et al., 2019). Longitudinal studies of epigenetic change and resilience still require future attention. Second, our method of measuring resilience was a self-report questionnaire, which may have resulted in biased resilience scores. Third, for complex psychological characteristics, multiple interacting factors play significant roles in developing and modulating resilience in an integrated way (Rutten et al., 2013). There are many factors that may affect resilience, and DNA methylation studies may not be able to comprehensively explore all relevant biological mechanisms of resilience (Smeeth et al., 2021). For example, genetics, epigenetics, developmental biology, environmental factors, psychosocial factors, neurochemicals, and functional neural networks (Wu et al., 2013; Niitsu et al., 2019). Fourth, the identification of biological mechanisms is challenging because of the paucity of data from epigenetic studies in peripheral tissues and CNS tissue and the possible bias of respective methylation signatures (Walton et al., 2016). Fifth, because this is a cross-sectional study, it can only be established that DNA methylation signatures are related to resilience, while possibly pointing to biological mechanisms. The longitudinal cohort study may be conducted in the future for exploring potential causality. Finally, all participants were Taiwanese (Han Chinese descents), so this is not necessarily applicable to other races.

In this study, we produced an LR methylation risk score from three methylation signatures to estimate individualized outcomes for LR identification. The methylation risk score model of our study is primarily used to identify whether individuals have low resilience risk. The accuracy of the discriminative model between LR and HR was 72.3% (logistic regression) and 87.1% (SVM), suggesting that this risk score model may be suitable in application of the psychological field. Although the results of classification are good, high dimensional data with small number of samples may lead to misclassification and biased discriminators. A larger sample size study data can improve the robustness of discriminative models. Furthermore, the resilience trait is related to many factors, it can only be preliminarily concluded that epigenetics may be related to resilience in the current study. But its explanatory power and the actual mechanism still need further study. In the future, we also expect that other variables can be added into the risk scoring model, such as environmental factors, genetic variation, different age groups, etc. We believe that this may have a higher discrimination ability in identifying low resilience groups. Further, we may also be able to examine the association of low resilience groups with mental illness states in the future, which may help prevent mental health deterioration. If we can use our risk scoring model well to find low resilience risk groups early on, we might be able to conduct early intervention to help improve mental health.

## Conclusion

Our findings support that evidence of a link between resilience and DNA methylation in peripheral blood and may provide further understanding of epigenetics in resilience traits. We also established the methylation risk score model using the methylation markers that may be useful in psychological practice or research. Our findings may be helpful in the development of psychosocial and psychological interventions for enhancing resilience and mitigating stress and adverse effect, and thus in improving mental health care.

## Data Availability

The original contributions presented in the study are included in the article/Supplementary Material, further inquiries can be directed to the corresponding author. The data are not publicly available due to ethical and privacy restrictions.
