# Supplementary material for: DNA methylation signature of psychological resilience in young adults: Constructing a methylation risk score using a machine learning method
Source: Front Genet. 2023 Jan 12;13:1046700. doi: 10.3389/fgene.2022.1046700 (PMC9877348; doi:10.3389/fgene.2022.1046700)
Supplement: Supplementary file 1 [file Table1.DOCX]

Supplementary Material

Supplementary Table 1 Primer sequences of methylation probes and gene identification in the validation set of this study.

| Probe ID | Gene | Chromosome location | Target sequence for primer of methylation probe |
| --- | --- | --- | --- |
| cg18565204 | *AARS* | chr16: 76075821-76075946 | CTACCACTGGAGTCCAAATGGTAATTGTACTTTGGGGAATCATCTGTGACCTCCAGACCC[CG]TGCCCGGAGCTCTTCGATAGCGTAAATGTCCAGCATAATGAGGTCTTCCCCACCAGCTCC |
| cg17682313 | *FBXW7* | chr4:153447805-153449689 | GAAGCCAAGAAATTATTGAATTATGAATTACTGAATGCATATTATGAATGTATTTTATTA[CG]TAAATGAGAAGTAAACTAGCTGCCTGTTGCTGACTCCAGGTAGACCTGTTTTCAACCATT |
| cg23024343 | *COG5* | chr7:107204114-107204797 | TACTATGCATCTGTGCTCACCAAGCCTATCAATCCGATACTCTGTCATTGGCCAAATCCC[CG]ATATAGTGAATCAAAGTTTCTGCTGGAAATGCTTAATTCCTCAGGAATGTTGGGGGTGGA |
| cg05194426 | *CYP2E1* | chr10:135341255-135342561 | ATCTAAAAATTAAATGAGCTGATAAAGAACGCCGTCAGCACAGAGCAGACGCTGGGTGTT[CG]CGCTCTTGAGCGTGCGCTCTGCGGGGCGCGGGCTGGTGGCGGGCGGGGGTCGCCGGCTCC |
| cg25755428 | *MRI1* | chr19:13875044-13875951 | GGCGGCTCACTCCGTTCGGGCTTGGCAGGAGTCGTGGAGTGGGTTCGGCCACGTGGAATC[CG]CGTCCTGGGAACCCGTGGAATCCGCGTCCTGGGAACCCGTGGAATCCGCCTCCTGGGAAC |
| cg23400446 | *CYP2E1* | chr10:135341255-135342561 | GGCTTCGTCCACCGAGGTCCCCTCACCCACGCTGAGGCGTCGGAAGCTGCGGACACTGCT[CG]CTTCAGGGCTTTGCTCAGCTGCAGCTGGTGACCTCCAGAGAGGGAGTCTCTGATGTCCCG |
| cg07167608 | *LINC01107* | chr2:239431185-239432537 | CCGGTCACATGGCTCCTCATGGCAGAGCTGGGATTTTCATCATTCAGCAAGTGTCTGCTG[CG]TCCCCATCTGGGCCCAGCCCTGAATTCAGAGCCAGTGCCACAGAAGCCAACCAGGCAAGC |
| cg03013609 | *LGALS8* | chr1:236701383-236704859 | CCTTGATGTCAGTTCCATCTCTGGCTTCATGGAGTGTCTTGTACCTAGCGTGTATGTGTA[CG]GTTGAATTTGGTCCCAGAAGCTTACACCTGCTGGCCCTCTGGCCTGTGGAGCTTGCCCAC |
| cg00321709 | *CYP2E1* | chr10:135341255-135342561 | CCCTCTGGGTTCTCTAGAGCAACAGCAATACCCGCCCGGCAGGGGTGTGGCTTAGAGCCC[CG]CACCTCCTCGCCGCGCGGCGGGCCTGACTTCTAGCCACGGGTCTCCGCAGTTGGCCCAGC |

Supplementary Table 2 Descriptive statistics of methylation level with LR and HR in the validation set.

|  | Low resilience individuals | | High resilience individuals | | P-value | FDR |
| --- | --- | --- | --- | --- | --- | --- |
|  | (n=31) | | (n=31) | |  |  |
| Variables | Mean | SD | Mean | SD |  |  |
| cg18565204 (*AARS*) | 55.57 | 19.15 | 45.46 | 13.22 | 0.0199 ^a^ | 0.0597 ^c^ |
| cg17682313 (*FBXW7*) | 33.41 | 16.16 | 18.94 | 14.14 | 0.0004 ^a^ | 0.0036 ^b^ |
| cg23024343 (*COG5*) | 26.50 | 24.40 | 26.27 | 20.39 | 0.9669 | 0.9669 |
| cg05194426 (*CYP2E1*) | 18.25 | 23.52 | 14.63 | 15.19 | 0.4781 | 0.7172 |
| cg25755428 (*MRI1*) | 49.12 | 45.97 | 42.15 | 38.47 | 0.5187 | 0.6669 |
| cg23400446 (*CYP2E1*) | 37.87 | 42.84 | 25.75 | 29.39 | 0.2025 | 0.4556 |
| cg07167608 (*LINC01107*) | 39.47 | 11.32 | 31.28 | 10.80 | 0.0050 ^a^ | 0.0225 ^b^ |
| cg03013609 (*LGALS8*) | 11.42 | 14.19 | 10.89 | 16.49 | 0.8934 | 1.0000 |
| cg00321709 (*CYP2E1*) | 23.42 | 26.75 | 18.84 | 19.55 | 0.4419 | 0.7954 |

LR, low resilience; HR, high resilience; FDR, false discovery rate.

^a^ significant difference between LR and HR, P < 0.05.

^b^ significant difference between LR and HR, FDR < 0.05.

^c^ marginal significant difference between LR and HR, FDR < 0.06.

Supplementary Table 3 The result of methylation level with CD-RISC score using linear regression in the validation set.

| Variables | Beta | SD | 95% CI | P-value |
| --- | --- | --- | --- | --- |
| **cg18565204 (*AARS*)** | -0.23 | 0.11 | -0.45, -0.00 | **0.0476^a^** |
| **cg17682313 (*FBXW7*)** | -0.40 | 0.11 | -0.62, -0.18 | **0.0004^a^** |
| cg23024343 (*COG5*) | 0.00 | 0.09 | -0.18, 0.18 | 0.9970 |
| cg05194426 (*CYP2E1*) | -0.02 | 0.10 | -0.22, 0.18 | 0.8577 |
| cg25755428 (*MRI1*) | -0.05 | 0.05 | -0.14, 0.05 | 0.3382 |
| cg23400446 (*CYP2E1*) | -0.04 | 0.05 | -0.15, 0.06 | 0.4203 |
| **cg07167608 (*LINC01107*)** | -0.32 | 0.16 | -0.65, 0.00 | **0.0530^b^** |
| cg03013609 (*LGALS8*) | 0.06 | 0.13 | -0.20, 0.32 | 0.6672 |
| cg00321709 (*CYP2E1*) | -0.02 | 0.09 | -0.19, 0.15 | 0.8472 |

^a^ significant at P < 0.05.

^b^ marginal significant at P < 0.06.

Supplementary Table 4 The result of methylation level with CD-RISC score using linear regression for adjusted gender and age in the validation set.

| Variables | Beta | SD | 95% CI | P-value |
| --- | --- | --- | --- | --- |
| **cg18565204 (*AARS*)** | -0.25 | 0.12 | -0.48, -0.02 | **0.0370^a^** |
| **cg17682313 (*FBXW7*)** | -0.40 | 0.11 | -0.63, -0.18 | **0.0006^a^** |
| cg23024343 (*COG5*) | -0.00 | 0.09 | -0.19, 0.19 | 0.9713 |
| cg05194426 (*CYP2E1*) | -0.03 | 0.11 | -0.25, 0.19 | 0.8167 |
| cg25755428 (*MRI1*) | -0.05 | 0.05 | -0.14, 0.05 | 0.3413 |
| cg23400446 (*CYP2E1*) | -0.05 | 0.06 | -0.16, 0.07 | 0.4207 |
| **cg07167608 (*LINC01107*)** | -0.34 | 0.17 | -0.67, 0.01 | **0.0532^b^** |
| cg03013609 (*LGALS8*) | 0.05 | 0.14 | -0.23, 0.33 | 0.7212 |
| cg00321709 (*CYP2E1*) | -0.02 | 0.09 | -0.20, 0.16 | 0.8514 |

^a^ significant at P < 0.05.

^b^ marginal significant at P < 0.06.

^
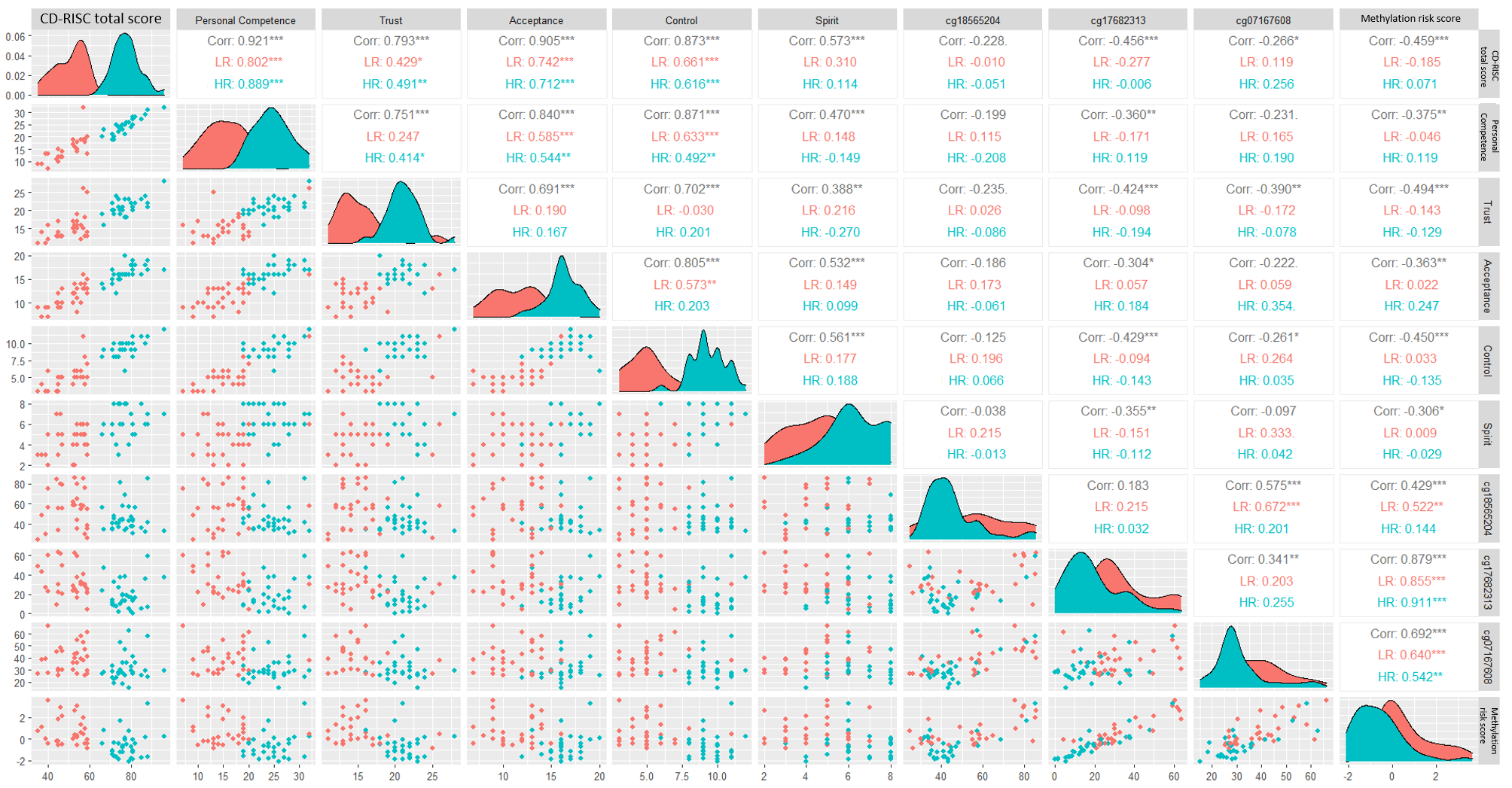
^

Supplementary Figure 1 Correlation plot of CD-RISC scores and methylation level in LR and HR groups.

P-value estimated from Spearman’s Rank-Order Correlation test. *** P < 0.001; ** P < 0.01; * P < 0.05.


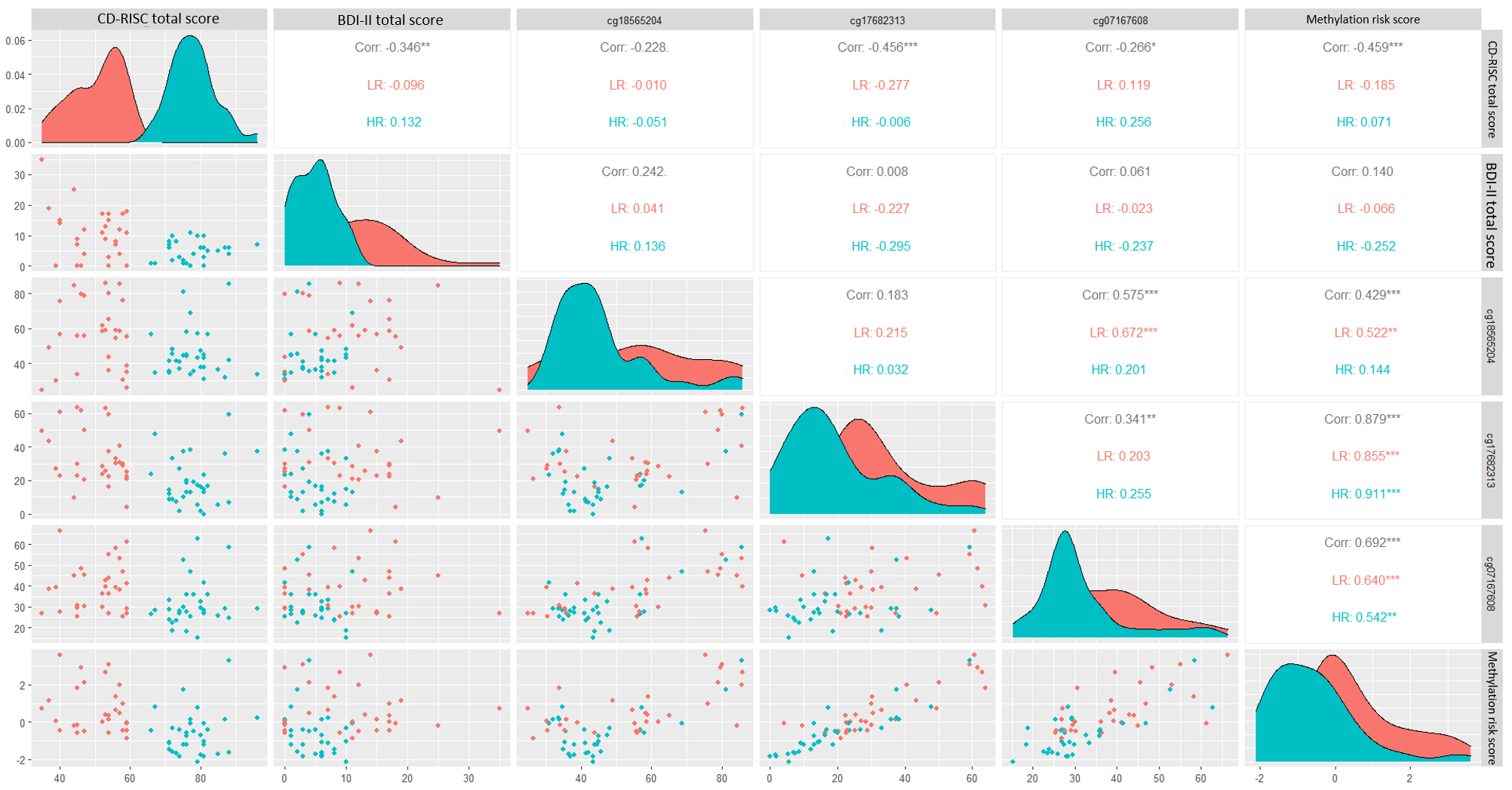


Supplementary Figure 2 Correlation plot of CD-RISC score, BDI-II scores and methylation probes in LR and HR.

P-value estimated from Spearman’s Rank-Order Correlation test. *** P < 0.001; ** P < 0.01; * P < 0.05.


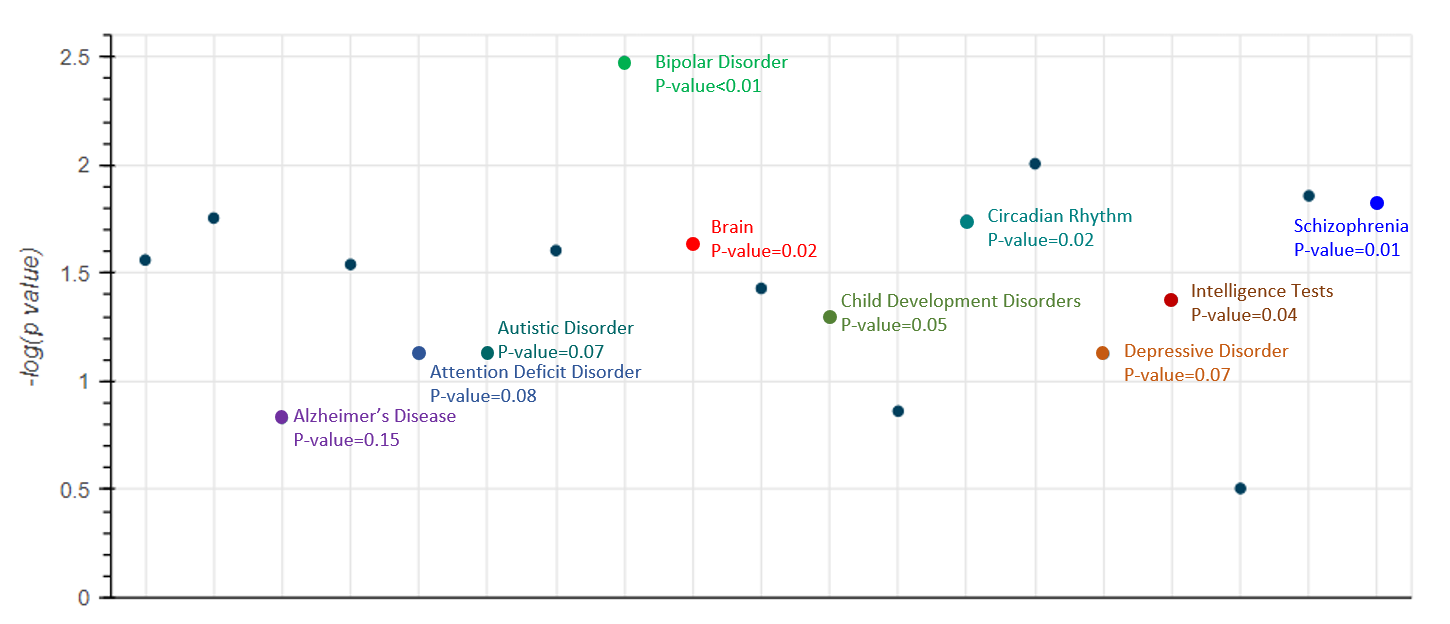


Supplementary Figure 3 Manhattan plot of enrichment analysis based on three resilience-associated methylation probes (methylation risk score) of the validation set using PhenGenI Association 2021 database.
